# Association between neutrophil extracellular traps, the Von Willebrand factor axis, and clinical outcome in stable coronary artery disease

**DOI:** 10.3389/fcvm.2026.1759117

**Published:** 2026-02-26

**Authors:** Vibeke Bratseth, Kristine M. Kindberg, Ellen M. K. Warlo, Alf-Åge R. Pettersen, Trine B. Opstad, Miriam S. Langseth, Ragnhild Helseth, Ida G. Lunde

**Affiliations:** 1 Oslo Center for Clinical Heart Research, Department of Cardiology Ullevål, Oslo University Hospital, Oslo, Norway; 2 Institute of Clinical Medicine, Faculty of Medicine, University of Oslo, Oslo, Norway; 3 Department of Medicine, Vestre Viken HF, Ringerike Hospital, Hønefoss, Norway; 4 Department of Cardiology Rikshospitalet, Oslo University Hospital, Oslo, Norway; 5 KG Jebsen Center for Cardiac Biomarkers, Campus Ahus, University of Oslo, Oslo, Norway

**Keywords:** ADAMTS13, atherosclerosis, citrullinated histone H3 (CitH_3_), endothelial dysfunction, NETs, thromboinflammation, VWF

## Abstract

**Introduction:**

Coronary artery disease (CAD) is the clinical manifestation of atherosclerosis, an inflammatory disorder of the coronary arteries, characterized by endothelial dysfunction, lipid accumulation, immune activation, and formation of atherosclerotic plaques. Despite management of conventional risk factors, CAD is a progressive disease, and may develop vulnerable lesions prone to rupture, causing atherothrombosis and acute coronary syndrome (ACS). Neutrophil extracellular traps (NETs), composed of chromatin and proteases, have been implicated in vascular inflammation and thrombosis, while the von Willebrand Factor (VWF)-ADAMTS13 (a disintegrin and metalloproteinase with thrombospondin type 1 motifs, member 13) axis plays a central role in platelet-mediated thrombus formation. Evidence suggest that NETs may interact with VWF to amplify thromboinflammation. The clinical relevance of this interplay and the prognostic utility of the NETs marker citrullinated histone H_3_ (CitH_3_) in CAD remains unclear.

**Aims:**

In stable CAD patients we aimed to 1) examine associations between CitH_3,_ cardiovascular risk factors, and clinical outcome, 2) explore potential interactions between NETs and the VWF-ADAMTS13 axis, and 3) evaluate the predictive value of combined biomarker profiles.

**Methods:**

Between 2003 and 2010, patients with angiographically verified symptomatic CAD (*n* = 1,000) were enrolled in the Aspirin Nonresponsiveness and Clopidogrel Endpoint Trial (ASCET) (NCT00222261). The primary composite endpoint (*n* = 73) at two-year follow-up comprised non-hemorrhagic stroke (*n* = 28), myocardial infarction (*n* = 36) and death (*n* = 9). Analyses were performed on baseline blood samples.

**Results:**

CitH_3_ levels were similar between patients with and without endpoints. CitH_3_ correlated with neutrophil count (*r* = 0.221, *p* < 0.001) and was higher in younger patients (<62 years) and in those with BMI above mean (>27.4 kg/m^2^). CitH_3_ alone did not predict clinical outcome. However, patients with high VWF, low ADAMTS13, and elevated NETs biomarkers had increased odds of reaching the composite endpoint (adjusted odds ratio 3.14 and 3.68). This subgroup also exhibited higher leukocyte counts and high-sensitivity C-reactive protein.

**Conclusion:**

CitH_3_ alone was not predictive of adverse events in stable CAD. However, combined extreme levels of thromboinflammatory biomarkers VWF, ADAMTS13 and NETs, identified patients at higher risk of adverse events. These findings suggest that integrated thromboinflammatory biomarker profiles may improve risk stratification in stable CAD and warrant validation in independent cohorts.

## 1 Introduction

Coronary artery disease (CAD) is a dynamic and chronic inflammatory disease in the epicardial arteries that is driven by atherosclerosis. Endothelial dysfunction, lipid accumulation, and dysregulated immune responses contribute to progressive plaque formation and increase the risk of atherothrombotic complications (1–3). These pathophysiological processes collectively impair myocardial perfusion and oxygen supply and may lead to symptoms like angina and dyspnea (4, 5). Timely diagnosis and appropriate treatment are essential to prevent the progression of CAD to acute coronary syndrome (ACS). However, management of conventional cardiovascular risk factors does not hinder CAD from progressing in all patients, and identifying high-risk individuals is crucial and part of precision-medicine efforts (6–9).

Neutrophils and neutrophil extracellular traps (NETs) play an increasingly recognized role in driving atherothrombosis (10). Beyond classical phagocytotic functions, activated neutrophils release NETs i.e., networks of decondensed chromatin decorated with citrullinated histones and granule proteins (11). In response to cardiovascular risk factors, such as oxidized low-density lipoproteins (LDL), cholesterol crystals, cytokines, and activated platelets (12), NETs have been shown to promote endothelial injury, platelet activation, and atherogenesis, thereby directly linking innate immunity to thrombosis (13–15). NETs are furthermore suggested to interact with key prothrombotic pathways including the Von Willebrand factor (VWF) and a disintegrin and metalloproteinase with thrombospondin type 1 motifs, member 13 (ADAMTS13) axis (16). The function and activity of VWF depend on its size, which is regulated by the metalloprotease ADAMTS13. VWF is a multimeric glycoprotein important for platelet adhesion and activation. It is stored in Weibel-Palade Bodies (WPBs) of endothelial cells and in platelet *α*-granules. Once secreted, the majority of VWF circulates in plasma while a proportion is associated with the luminal surface of endothelial cells. Low levels of ADAMTS13 as well as high VWF/ADAMTS13 ratio have been associated with coronary heart disease and cardiovascular events (17–19). Recently, a more distinct role of VWF has been found when it comes to recruitment of neutrophils (20). At sites of vascular injury, dysregulation of VWF and disturbances in the VWF-ADAMTS13 axis may initiate a self-perpetuating cycle in which platelets, neutrophils, and NETs collectively amplify inflammation and promote thrombus formation, i.e., thromboinflammation (21). NETs have been shown to bind directly to VWF and may provide a scaffold for coagulation factors (16, 22, 23). Through these mechanisms, NETs may contribute to the transition from CAD to clinical manifestations (24–26). The clinical relevance of immune activation in CAD patients was recently underscored by the landmark CANTOS trial, which demonstrated that selective Il-1β inhibition with canakinumab significantly reduced recurrent cardiovascular events in such patients (27). Together, these insights highlight the importance of understanding thromboinflammatory mechanisms for improving risk stratification and future therapeutic strategies.

Circulating NETs levels are quantified using assays targeting specific components of NETs, such as myeloperoxidase (MPO)-DNA complexes, double-stranded (ds)-DNA, neutrophil elastase and citrullinated histone H_3_ (CitH_3_). CitH_3_ is considered a hallmark of NETosis, reflecting chromatin decondensation and NETs release (28). Accumulating evidence supports the role of NETs in ACS and biomarkers of NETs may predict clinical outcome in these patients (29–31). Although we have previously shown an association between dsDNA and clinical outcome in stable CAD (32), the predictive power of NETs biomarkers in CAD is unclear (33). Furthermore, the combined contribution of NETs and the VWF-ADAMTS13 axis to the progression of CAD and prediction of cardiovascular events are not fully understood. A deeper understanding of this interplay is needed, as thromboinflammation remains an unaddressed target in clinical practice (34), and identifying patients with this inflammatory-thrombotic phenotype is warranted. Thus, in patients with stable CAD, we here aimed to explore 1) associations between CitH_3_, cardiovascular risk factors and clinical outcome and 2) associations between NETs and the VWF-ADAMTS13 axis, and 3) combined predictive value of NETs and VWF-ADAMTS13 levels.

## 2 Materials and methods

### 2.1 Study population

The current observational investigation is a substudy of the ASCET trial (Aspirin Nonresponsiveness and Clopidogrel Endpoint Trial) conducted at the Department of Cardiology Ullevål, Oslo University Hospital, Oslo, Norway. Between March 2003 and July 2010, patients with stable symptomatic CAD were enrolled in the ASCET trial (*n* = 1,000) (35). Eligibility screening was performed after coronary angiography had confirmed the presence of CAD. Patients with ACS and those treated with percutaneous coronary intervention (PCI) were eligible for enrolment after completing the guideline-recommended duration of dual antiplatelet therapy, as determined by the treating physician in accordance with the European Society of Cardiology (ESC) Guidelines, typically one to 12 months after initial screening. In the ASCET trial, all patients were on antiplatelet monotherapy with aspirin at the time of blood sampling. Patients using oral anticoagulants were excluded. Upon inclusion, they were randomized to either continue aspirin therapy or a switch to clopidogrel. At the two-year follow-up, the primary endpoint was defined as the first occurrence of a composite event consisting of myocardial infarction (MI), non-hemorrhagic stroke, or all-cause mortality, and was documented as a binary outcome (yes/no). This definition differed slightly from the original composite endpoint in the ASCET trial, as the less reliable diagnosis of unstable angina was excluded (35). The ASCET trial was conducted prior to the introduction of high-sensitivity troponin assays. All participants gave written informed consent. The ASCET study was approved by the regional committees for medical and healthcare research ethics (REK ID# 209/02), was registered at https://www.clinicaltrials.gov (ID# NCT00222261), complied with the Declaration of Helsinki and Good Clinical Practice (GCP) procedures, and the continued use of the biobank was granted (REK ID# 2017/652).

### 2.2 Laboratory methods

Venous blood samples were drawn in a fasting condition before morning medication and prior to inclusion. Whole blood was allowed to clot for up to 1 h at room temperature and centrifuged at 2,500×*g* for 10 min. Citrated blood (0.129 M; 1:10 dilution) was kept on ice and processed within 30 min with centrifugation at 4 °C, 3,000×*g* for 20 min. Aliquots of serum and citrated plasma were stored at −80 °C until analysis to preserve stability. CitH_3_ was assessed in serum by a commercially available enzyme linked immunosorbent assay (ELISA) kit (Cayman Chemical, Ann Arbor, MI, USA, Cat nr 501620), according to the manufacturer's instructions. In brief, the assay employed a CitH_3_-specific capture antibody and a horseradish peroxidase (HRP)-conjugated detection antibody. Color development was achieved with tetramethylbenzidine (TMB), and absorbance was measured at 450 nm on a microplate reader (EPOCH2TS, BioTek Instruments, supplied by AH Diagnostics, Oslo, Norway). The concentrations were calculated from a standard curve generated with known CitH_3_ standards, and the inter-assay CV was 12.7%. The previously assessed biomarkers of NETs in the ASCET trial, i.e., dsDNA and MPO-DNA, were also analyzed in serum (32). In short, dsDNA was measured with use of the fluorescent nucleic acid stain Quant-iT PicoGreen (Invitrogen Ltd., Paisley, UK, Cat nr P7589) and detected by fluorometry (Fluoroskan Ascent, Thermo Fisher Scientific Oy, Vantaa, Finland). MPO-DNA complexes were identified using an ELISA technique, as described (36). Briefly, microtiter plates (Immulon 4HBX, flat bottom, Thermo Fisher Scientific, MA, USA, Cat nr 3855) were coated with anti-MPO monoclonal antibody (AbD Serotec, Hercules, CA, USA, Cat nr 0400-0002) and incubated overnight at 4 °C. After blocking with bovine serum albumin (BSA) 1%, patient serum and a peroxidase-labeled anti-DNA monoclonal antibody (Cell Death detection kit; Roche Diagnostics, Mannheim, Germany, Cat nr 11774425001) were added. Following incubation and washing, a peroxidase substrate was applied, and absorbance was measured after 40 min. Results were expressed as optical density units. The inter-assay CVs for dsDNA and MPO-DNA measurements were 7.0% and 10.5%, respectively. We have previously reported measurements of VWF, ADAMTS13 antigen (Ag), P-selectin, and high sensitivity (hs)-CRP (19, 35, 37). VWF, ADAMTS13 Ag, and P-selectin were analyzed in citrated plasma using the following commercially available ELISA kits: Asserachrom VWF Ag (Stago Diagnostica, Asnières, France, Cat nr 00492), ADAMTS13 Ag IMUBIND® (Sekisui Diagnostics GmbH, Pfungstadt, Germany, Cat nr 813) and P-selectin (R&D Systems Europe, Abingdon, Oxon, UK, Cat nr DPSE00). Hs-CRP was measured in serum (DRG Instruments, Marburg/Lahn, Germany, Cat nr EIA-3954). The inter-assay CVs for these assays were 6.3%, 8.7%, 7.2%, and 10.0%, respectively. Standard clinical chemistry analyses were conducted at the Department of Medical Biochemistry, Oslo University Hospital Ullevål using accredited methods.

### 2.3 Statistical analyses

Statistical analyses were performed with SPSS Inc., IL, USA version 29 and 30. Clinical characteristics were expressed as median (25th, 75th percentiles) or mean (±standard deviation) for continuous variables, according to normal or non-normal distribution-, and as numbers (percentages) for categorical variables. Group comparisons were performed with unpaired Student's *t*-test, Mann–Whitney *U* test and Chi-Square test, as appropriate. Groups were defined either by natural categories (e.g., sex) or by dichotomizing continuous variables at the median (above vs. below median) or between quartile Q1–Q3 and quartile Q4. Spearmans's rank correlation analyses were applied to measure the strength and direction of associations. Binary logistic regression analyses were used to estimate odds ratio (OR) with 95% confidence intervals (CIs) for the composite clinical endpoint. In the models, three combinations of thromboinflammatory markers including VWF and CitH_3_ in the highest quartile (Q4) and ADAMTS13 Ag in the lowest quartile (Q1) (combination I), VWF and dsDNA in Q4 and ADAMTS13 Ag in Q1 (combination II), and VWF and MPO-DNA in Q4 and ADAMTS13 Ag in Q1 (combination III) were evaluated. Sensitivity analysis was performed with VWF and ADAMTS13 Ag. Diabetes mellitus, previous MI, hypertension and hs-CRP were considered potential confounders. Significant associations were adjusted for age and sex, in addition to confounders with a significant contribution by multivariate logistic regression analyses. A *p*-value <0.05 was considered statistically significant. The Bonferroni method was used for multiple comparisons in the correlation analyses (*p* = 0.05/15 = 0.0033). This observational cohort study was reported in accordance with the STROBE (Strengthening the Reporting of Observational Studies in Epidemiology) guidelines. A completed STROBE cohort checklist is provided in the Supplementary Material.

## 3 Results

### 3.1 Study population

Baseline characteristics of the total stable CAD population (*n* = 1,000) are presented in Table 1, stratified by whether patients experienced a composite clinical endpoint (*n* = 73) or not (*n* = 927) at two-year follow-up. In brief, 21.8% were women, mean age was 62 years, and 96.8% were Caucasian. The average body mass index (BMI) was 27.4, 20% had diabetes, and more than half had hypertension (*n* = 556). Almost all patients used statins. Patients with the composite clinical endpoint registered at two-year follow-up, *n* = 73, more frequently had CVD, previous MI and diabetes. Of note, in the ASCET trial (35), the incidence of the composite clinical endpoint was similar between the aspirin and clopidogrel groups; therefore, the treatment arms were merged for this *post-hoc* analysis.

**Table 1:** Baseline characteristics and blood biomarker levels of the total stable CAD population.

Coronary artery disease (CAD) cohort	Total population (*n* = 1,000)	Primary composite endpoint at two-year follow-up: MI, non-hemorrhagic stroke and all-cause mortality		
		Yes (*n* = 73)	No (*n* = 927)	*p*-value
Baseline characteristics				
Age, mean (min, max) (years)	62 (36, 81)	64 (45, 80)	62 (36, 81)	0.074
Sex, female, *n* (%)	218 (21.8)	17 (7.8)	201 (92.2)	0.769
Caucasian, *n* (%)	968 (96.8)	72 (7.4)	896 (92.6)	0.306
Cardiovascular risk factors, *n* (%)				
Diabetes Mellitus	200 (20.0)	22 (30.1)	178 (19.2)	**0**.**036**
Hypertension	556 (55.6)	44 (60.3)	512 (55.3)	0.482
Current smokers	203 (20.3)	20 (27.4)	183 (19.8)	0.159
CVD	635 (63.5)	57 (78.1)	578 (62.4)	**0**.**011**
Previous MI	436 (43.6)	42 (57.5)	394 (42.6)	**0**.**019**
Diastolic blood pressure (mmHg)	82 ± 10	81 (±9)	82 (±10)	0.468
Systolic blood pressure (mmHg)	140 ± 19	140 (±19)	139 (±19)	0.844
Body mass index (kg/m^2^)	27.4 ± 3.7	27.3 (± 4.0)	27.4 ± (3.7)	0.742
Laboratory analyses (previously measured^1^)				
Total cholesterol, (mmol/L)	4.53 ± 0.95	4.51 (± 0.94)	4.53 (±0.95)	0.927
LDL cholesterol, (mmol/L)	2.53 ± 0.83	2.49 (±0.74)	2.53 (±0.84)	0.706
HDL cholesterol, (mmol/L)	1.34 ± 0.41	1.35 (±0.39)	1.34 (±0.41)	0.822
Triglycerides, (mmol/L)	1.31 (0.93, 1.84)	1.26 (0.94, 1.86)	1.31 (0.93, 1.82)	0.897
Leukocyte count (×10^9^/L)	6.20 (5.30, 7.40)	6.40 (5.50, 8.08)	6.20 (5.30, 7.40)	0.313
Neutrophil count (×10^9^/L)	3.40 (2.70, 4.20)	3.55 (2.80, 4.83)	3.40 (2.80, 4.83)	0.211
Platelet count (×10^9^/L)	228 (195, 264)	221 (181, 272)	229 (196, 264)	0.446
P-selectin (ng/mL)	30.00 (21.50,	29.80 (21.60, 38.80)	31.70 (20.71, 43.00)	0.461
Hs-CRP (mg/L)	2.24 (1.02, 4.14)	2.67 (1.21, 4.76)	2.22 (0.99, 4.07)	0.079
VWF Ag, (IU/mL)	1.06 (0.83, 1.33)	1.13 (0.92, 1.40)	1.05 (0.82, 1.33)	**0**.**032**
ADAMTS13 Ag, (ng/mL)	531 (461, 607)	506 (452, 572)	533 (462, 607)	0.147
Ratio VWF/ADAMTS13 Ag, ×10^−3^ IU/ng	1.99 (1.51, 2.65)	2.27 (1.67, 2.95)	1.97 (1.50, 2.64)	**0**.**012**
dsDNA, (ng/mL)	395 (360, 434)	407 (375, 449)	394 (360, 433)	0.059
MPO-DNA, (OD)	0.141 (0.127, 0.164)	0.141 (0.123, 0.157)	0.140 (0.126, 0.163)	0.633
Laboratory analysis (current study)				
CitH_3_, (ng/mL)	2.45 (1.56, 4.64)	2.08 (1.51, 4.29)	2.46 (1.58, 4.65)	0.050
Medication, %				
Statins	98.2	98.6	98.3	0.644
*Β*-blockers	75.5	75.0	75.9	0.982
Angiotensin-converting enzyme inhibitors	26.3	32.9	26.0	0.249
Angiotensin receptor blockers	23.9	31.5	23.5	0.159

Statistically significant *p*-values are bolded. The table presents baseline characteristics and blood biomarkers concentrations in the total ASCET population and according to having a clinical endpoint (yes) or not (no). For the current study, the NETs biomarker CitH_3_ was measured. Values are mean (±SD), median (25th, 75th percentiles) or numbers (proportions), as appropriate. For continuous data, two-sided independent Student's *t*-test with equal variances assumed and Mann–Whitney *U* test were used, according to distribution of data, to compare between the two clinical endpoints groups. Chi-Square test was used on categorical data. CVD includes previous MI, percutaneous coronary intervention, coronary artery bypass graft and non-hemorrhagic stroke. Diabetes mellitus includes both type 1- and type 2 diabetes mellitus.

CVD, cardiovascular disease; MI, myocardial infarction; LDL, low density lipoprotein; HDL, high density lipoprotein; hs-CRP, high-sensitivity C reactive protein; VWF, von Willebrand factor; ADAMTS13, a disintegrin and metalloproteinase with thrombospondin type 1 motifs; member 13; Ag, antigen; dsDNA, double-stranded DNA; MPO-DNA, myeloperoxidase-DNA and CitH_3_, citrullinated histone H_3_.

^1^ The conventional clinical chemistry analyses, i.e., cholesterol and cell counts were measured at the hospital's central laboratory. P-selectin, hs-CRP, VWF, ADAMTS13 Ag and the NETs biomarkers dsDNA and MPO-DNA were assessed with ELISAs at our research lab and are part of previous publications from our group.

### 3.2 Blood biomarker levels

Baseline levels of CitH_3,_ and the previously measured NETs biomarkers MPO-DNA and dsDNA did not differ significantly between stable CAD patients experiencing the composite clinical endpoint or not (Table 1). As expected, CitH_3_ was positively correlated with MPO-DNA and dsDNA, respectively (*r* = 0.291 and *r* = 0.146, *p* < 0.001, both), and correlated with neutrophil count (*r* = 0.221, *p* < 0.001).

We have previously reported significantly higher levels of VWF and VWF/ADAMTS13 Ag ratio in patients having a clinical endpoint (19), whereas neutrophil- and platelet count, as well as P-selectin and hs-CRP were similar in the two endpoint-groups (Table 1).

### 3.3 Citrullinated histone H_3_ according to cardiovascular risk factors and clinical endpoint

Patients younger than the mean age (<62 years) and those with BMI above the mean (>27.4 kg/m^2^) had significantly higher CitH_3_ levels (*p* = 0.046 and *p* = 0.022, respectively). No other cardiovascular risk factors were associated with CitH_3_ concentrations (Supplementary Table S1).

Among patients who experienced a clinical endpoint (*n* *=* 73; 36 MI, 28 non-hemorrhagic strokes, and 9 deaths), CitH_3_ levels tended to be lower compared to those without an endpoint (Table 1). However, when CitH_3_ was categorized into quartiles (Q1–3 ≤ 4.63 ng/mL vs. Q4 ≥ 4.64 ng/mL), univariate analysis showed no significant association with endpoint risk (OR: 0.70, 95% CI: 0.38, 1.28, *p* = 0.243; Supplementary Figure S1).

### 3.4 Correlations between circulating markers of NETs and the VWF-ADAMTS13 axis

Overall correlations between circulating markers of thromboinflammation are summarized in Table 2. Initial analysis demonstrated significant correlations between dsDNA and both ADAMTS13 Ag and the VWF/ADAMTS13 Ag ratio, however, these associations did not remain significant after Bonferroni correction. Subgroup analyses [patients with a clinical endpoint (*n* = 73), and those with previous MI (*n* = 36)], revealed no significant correlations (data not shown).

**Table 2:** Associations between circulating biomarkers of NETs and the VWF-ADAMTS13 axis.

Biomarkers	Spearman's rho		
	CitH_3_	MPO-DNA	dsDNA
VWF Ag	−0.017	−0.003	0.060
ADAMTS13 Ag	0.021	0.019	−0.072*
VWF/ADAMTS13 Ag	−0.018	−0.006	0.085**

VWF, von Willebrand factor; ADAMTS13, a disintegrin and metalloproteinase with thrombospondin type 1 motifs; member 13; Ag, antigen; CitH_3_, citrullinated histone H_3_; MPO-DNA, myeloperoxidase-DNA; dsDNA, double-stranded DNA.

Spearman's rho between biomarkers of the VWF-ADAMTS13 axis and NETs in blood. Level of significance: *0.05 and **0.01. After correction for multiple comparisons, the statistically significant correlations disappeared.

### 3.5 Combined high VWF and low ADAMTS13 Ag with NETs biomarkers predict clinical outcome and reflect inflammatory profile

We first evaluated the predictive value of combining Q4 of VWF with Q1 of ADAMTS13 Ag for the composite clinical endpoint (sensitivity analysis, Supplementary Table S2). Subsequently, we incorporated NETs biomarkers to create three combinations:

Combination I: VWF ≥1.33 IU/mL, ADAMTS13 Ag <461 ng/mL, and CitH_3_ ≥ 4.64 ng/mL
Combination II: VWF ≥1.33 IU/mL, ADAMTS13 Ag <461 ng/mL, and dsDNA ≥434 ng/mL
Combination III: VWF ≥1.33 IU/mL, ADAMTS13 Ag <461 ng/mL, and MPO-DNA ≥0.164 OD

**Figure 1:**
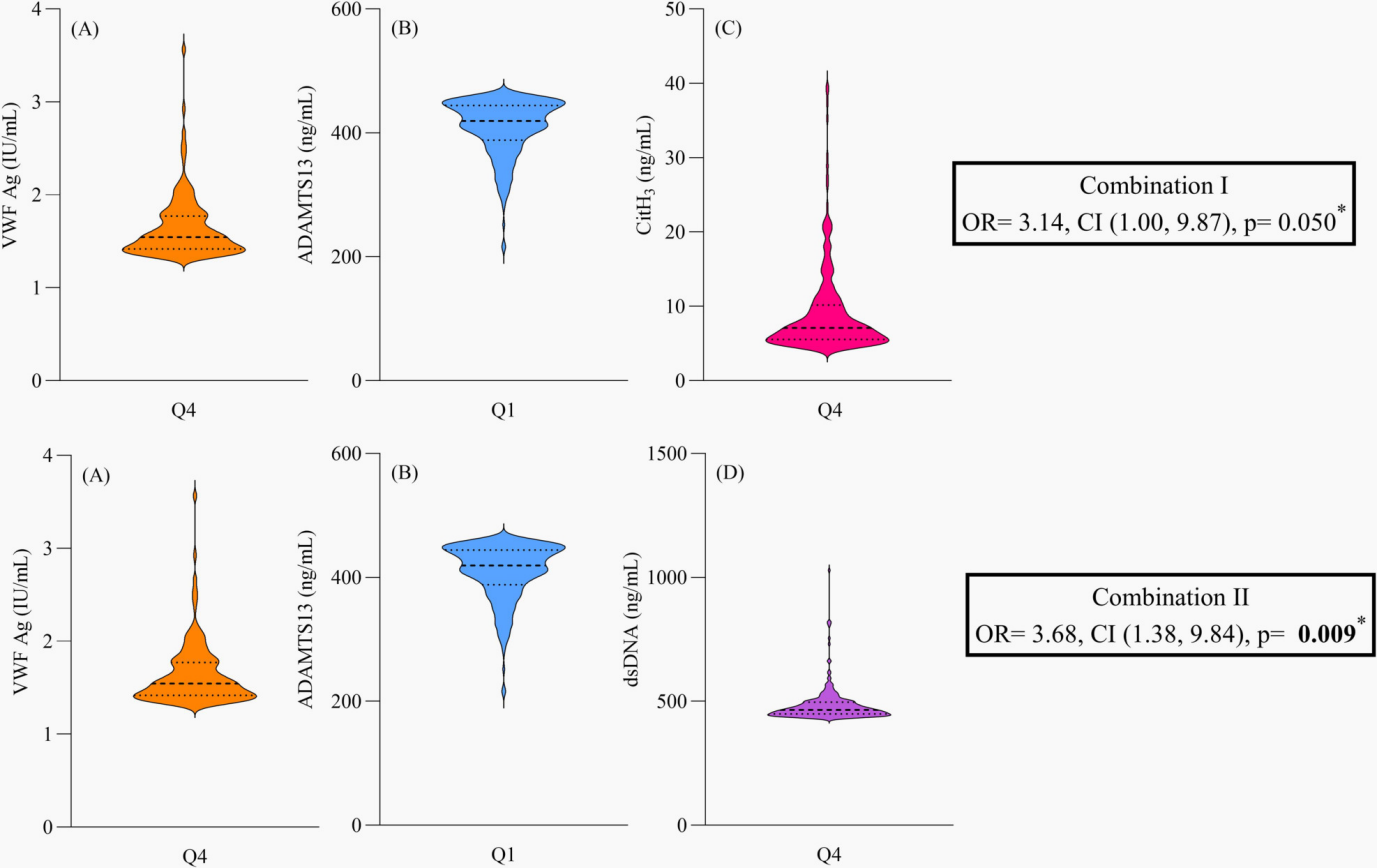
Biomarkers of the combined thromboinflammatory profile and the risk of clinical outcome. Combination I included VWF Ag in Q4 **(A)**, ADAMTS13 Ag in Q1 **(B)** and CitH_3_ in Q4 **(C)** with an OR = 3.14, CI (1.00, 9.87), *p* = 0.050. Combination II included VWF Ag in Q4 **(A)**, ADAMTS13 Ag in Q1 **(B)** and dsDNA in Q4 **(D)** with an OR = 3.68, CI (1.38, 9.84), *p* = **0.009**. Bolded value means it is below the significance level of 0.05. *Adjusted for age, sex, diabetes mellitus and previous myocardial infarction. VWF, Von Willebrand Factor, Ag, antigen, ADAMTS13, a disintegrin and metalloprotease with thrombospondin type 1 motif member 13, CitH_3_, citrullinated histone H_3_, dsDNA, double stranded DNA, Q4, highest quartile, Q1, lowest quartile, OR, odds ratio, CI, confidence interval.

Combination I (CitH_3_) and Combination II (dsDNA) were associated with an increased odds of reaching the composite endpoint [OR = 3.30 (CI = 1.07, 10.14), *p* = 0.037 and OR = 4.52 (CI = 1.73, 11.77), *p* = 0.002, respectively], whereas Combination III (MPO-DNA) showed no significant effect. Univariate- and multivariate analyses for the significant combinations, and the effect measure of separate clinical characteristics on the clinical outcome, are presented in Table 3. The biomarker distribution for these combinations and adjusted ORs are illustrated in Figure 1. Importantly, patients with Combination I or II exhibited significantly higher levels of circulating leukocytes, neutrophils, and hs-CRP compared with the remaining cohort (Table 4), indicating a pronounced inflammatory profile in these high-risk patients.

**Table 3:** Logistic regression analyses with combined biomarkers of thromboinflammation and risk of primary composite endpoint in stable CAD patients.

Univariate	OR	CI	*p* value	Multivariate*	OR	CI	*p* value
VWFQ4/ADAMTS13AgQ1/CitH_3_Q4, Combination I	3.30	1.07, 10.14	**0**.**037**		3.14	1.00, 9.87	0.050
VWFQ4/ADAMTS13AgQ1/dsDNAQ4, Combination II	4.52	1.73, 11.77	**0**.**002**		3.68	1.38, 9.84	**0**.**009**
Age	1.03	1.00, 1.05	0.074				
Sex	1.10	0.62, 1.93	0.749				
Diabetes Mellitus	1.82	1.07, 3.07	**0**.**026**				
Previous myocardial infarction	1.83	1.13, 2.96	**0**.**014**				
Hypertension	1.23	0.75, 2.00	0.410				
hs-CRP	1.05	1.00, 1.10	0.058				

OR, odds ratio; CI, confidence interval; VWF, von Willebrand factor; ADAMTS13, Ag a disintegrin and metalloproteinase with thrombospondin type 1 motifs; member 13 antigen; CitH_3_, citrullinated histone H_3_; dsDNA, double-stranded DNA; hs-CRP, high-sensitivity C reactive protein.

* Adjusted for age, sex, diabetes mellitus and previous myocardial infarction. Combination I (*n* = 20) and Combination II (*n* = 24).

Statistically significant *p*-values are bolded.

**Table 4:** Clinical characteristics in patients with biomarker combination I or II vs. the remaining study population.

Characteristics	Combination I			Combination II		
	Remaining study population (*n* = 949)	Q4 VWF Q1 ADAMTS13Ag and Q4 CitH3 (*n* = 19)	*p*	Remaining study population (*n* = 948)	Q4 VWF Q1 ADAMTS13Ag and Q4 dsDNA (*n* = 20)	*p*
Leukocyte count (×10^9^/L)	6.2 (5.3, 7.4)	7.2 (6.3, 9.6)	**<0.001**	6.2 (5.3, 7.4)	7.2 (6.3, 8.5)	**0** **.** **002**
Neutrophil count (×10^9^/L)	3.4 (2.7, 4.2)	4.7 (3.5, 6.6)	**<0.001**	3.4 (2.7, 4.2)	4.3 (3.0, 5.4)	**0** **.** **002**
Platelet count (×10^9^/L)	226 (194, 263)	260 (225, 318)	**0** **.** **004**	227 (195, 264)	232 (192, 281)	0.458
P-selectin (ng/mL)	30 (22, 39)	30 (23, 39)	0.741	30 (22, 39)	31 (25, 42)	0.420
hs-CRP (mg/L)	2.22 (1.04, 4.10)	3.06 (2.32, 8.80)	**0** **.** **037**	2.22 (1.01, 4.06)	3.32 (2.34, 9.38)	**<0.001**
Age (years)	62 ± 9	64 ± 10	0.347	62 ± 9	64 ± 10	0.355
Sex, female *n* (%)	216 (21.6)	2 (0.2)	0.276	214 (21.4)	4 (0.4)	0.802
BMI (kg/m^2^)	27.4 ± 3.7	26.4 ± 3.9	0.220	27.4 ± 3.7	26.1 ± 3.3	0.083
DBP (mmHg)	82 ± 10	80 ± 11	0.237	82 ± 10	79 ± 12	0.142
SBP (mmHg)	140 ± 19	139 ± 22	0.861	140 ± 19	134 ± 19	0.155
Hypertension *n* (%)	544 (54.5)	12 (1.2)	0.821	545 (54.6)	11 (1.1)	0.406

Q, quartile; VWF, von Willebrand factor; ADAMTS13, Ag a disintegrin and metalloproteinase with thrombospondin type 1 motifs member 13 antigen; CitH_3_, citrullinated histone H_3_; dsDNA, double-stranded DNA; hs-CRP, high-sensitivity C reactive protein; BMI, body mass index; DBP, diastolic blood pressure; SBP, systolic blood pressure.

Statistically significant *p*-values are bolded. Values are given as mean (±SD), median (25th, 75th percentiles) or numbers (proportions) as appropriate. Two-sided independent Student's *t*-test with equal variances assumed and Mann–Whitney *U* test were used for group comparisons for continuous data according to distribution of data. Fisher's test was used on categorical data.

## 4 Discussion

In stable CAD patients, higher circulating levels of the NETs biomarker CitH_3_ were associated with younger age (<62 years) and being overweight. NETs biomarkers and the VWF-ADAMTS13 axis were not significantly correlated, and CitH_3_ alone did not predict future clinical events. However, CitH_3_ and dsDNA, respectively, in combination with low circulating levels of ADAMTS13 Ag and high levels of VWF, was predictive of MI, non-hemorrhagic stroke, and all-cause mortality.

### 4.1 Correlation between NETs and the VWF-ADAMTS13 axis

Circulating levels of the measured NETs biomarkers (CitH_3_, MPO-DNA and dsDNA) were not significantly associated with the VWF-ADAMTS13 axis in our relatively large cohort of stable CAD patients. To date, no studies have specifically examined this potential interplay. In contrast to our findings, a weak relationship among extracellular DNA, NETs biomarkers, and VWF was observed in patients with suspected CAD referred to cardiac computed tomography angiography (CCTA) (38). In a small study in patients with coronavirus disease (COVID 2019) vs. healthy controls, a strong correlation between NETs and VWF was found at high vs. low levels of cell-damage [measured with cell-free DNA (cfDNA)] (39). Despite guideline-directed medical therapy, patients with CAD continue to have a clinically relevant residual atherothrombotic risk driven by persistent platelet hyperreactivity, ongoing systemic inflammation, and existing atherosclerotic plaques (9). Therefore, one may hypothesize that the lack of association between circulating NETs biomarkers and VWF in stable CAD patients could reflect either the binding of NETs to VWF or sequestration of NETs components within microvascular thrombi (10, 40, 41). Such mechanisms would reduce their availability as circulating markers. Supporting this, a positive correlation between VWF and DNA has been demonstrated in arterial thrombi from patients with CAD and peripheral artery disease (42). Nevertheless, the lack of association in the current cohort of stable CAD patients may be ascribed to optimal treatment according to current guidelines. All patients were taking aspirin and 98% were using statins. Antiplatelet treatment has been shown to attenuate NETosis (43, 44).

### 4.2 Markers of thromboinflammation and clinical outcome

When we explored CitH_3_ as a single biomarker of NETs, it was based on the hypothesis that measuring NETosis at a single time point might reflect a state of chronically increased NETs release, however, it had no predictive value for the composite endpoint during the two-year follow-up. Limited data exists on the predictive role of CitH_3_ and clinical outcomes in CAD. In the previously mentioned study in patients with suspected CAD (*n* = 282), dsDNA, MPO-DNA and nucleosomes predicted the severity of the disease and the occurrence of MACEs (*n* = 27, 9.7%) after a median of 545 days follow-up (38). Consistent with our findings, no association with citrullinated histone H4, another NET associated protein, was detected. The evidence for NETs biomarkers in predicting MACE and mortality in STEMI patients has been more established (45, 46). Recently, CitH_3_-DNA measured in the acute phase was found to be elevated and independently associated with mortality after 30 days and MACE after one year, despite a markedly reduction in CitH_3_-DNA concentration at six months (47). Its predictive value was enhanced when combined with CRP, reinforcing the concept of thromboinflammation. The type of NETs released have been suggested to depend on the activating stimuli, which may explain the varying results across clinical manifestations of CAD (48).

We have previously reported an association between circulating dsDNA and MACE in the present population (32). The contrasting results regarding specific NETs biomarkers, may also be attributable to the different methods applied, as well as their specificity for NETs. DsDNA also represent systemic inflammation and cell-death in general. Moreover, the lack of association between CitH_3_ and MACE may be explained by a lower number of events in the current study, due to exclusion of unstable angina which was originally included in the composite endpoint definition.

Consistent with our observation of higher NETs levels in overweight stable CAD patients, NETs biomarkers were found to associate with BMI and hypertension in another CAD cohort (49). In patients with coronary stenosis compared to matched controls, higher levels of neutrophils were found and NETs biomarkers predicted the degree of stenosis (49). These findings are plausibly accounted for by the heightened state of chronic low-grade inflammation, endothelial dysfunction and atherogenesis associated with cardiovascular risk factors, driving the release of NETs (1).

Interestingly, in a subset of our stable CAD patients, we identified a distinctive biomarker profile characterized by low ADAMTS13 Ag and high VWF levels, in combination with high circulating levels of CitH_3_ or dsDNA. These combined profiles independently predicted the incidence of new MI, non-hemorrhagic stroke, and all-cause mortality. To the best of our knowledge, this is the first study to demonstrate the utility of a combined thromboinflammatory biomarker profile in predicting adverse clinical outcomes in stable CAD. We speculate that such an imbalance in the VWF-NETs axis may cause a deleterious cycle with presence of ultralong VWF multimers, increased platelet- and neutrophil activation, NETosis, endothelial dysfunction and thrombosis. In accordance, we also found significantly higher levels of leukocytes and neutrophils in this subset of patients. In those with the biomarker profile low ADAMTS13 Ag, high VWF and high CitH_3_, we found significantly higher concentration of the inflammatory marker hs-CRP and platelet count, but not markers of platelet activation. P-selectin has been shown to promote NETs in mice, and increased levels have been found in patients with cardiovascular diseases, and to be associated with an elevated risk of MI, stroke and cardiovascular death (50–52). The combinations were superior to the single NET biomarkers and may enlighten mechanisms behind progression of disease and the increased thrombotic risk. In patients with COVID 2019, thrombotic microangiopathy and heart failure, NETs are already well-known to drive disease progression (26, 53, 54). Based on our findings, we suggest that adding NETs to the biomarker profile enhance the predictive power for experiencing new clinical events in patients with stable CAD. Unlike with dsDNA and CitH_3_, the biomarker profile with low ADAMTS13 Ag, high VWF and high MPO-DNA did not predict new clinical events in our cohort. This may be attributed to the heterogeneity of neutrophil subtypes and the diversity of NETs-inducing stimuli, which can influence the composition and functional properties of the NETs released (48).

### 4.3 Limitations

Although CitH_3_ is considered a specific biomarker of NETs, the detection of enzymatically citrullinated H_3_ proteins has been impeded by variability in enzyme production and stability as well as poor antibody specificity in commercially available methods (55). Future research should focus on developing robust assays for NETs to enhance clinical utility. Assessing circulating levels of surrogate biomarkers may not reflect, and therefore limit, the understanding of vascular and local activity. This retrospective cohort study included small subgroups and limited endpoint numbers; therefore, the findings should be interpreted with caution and confirmed in a larger, prospective studies.

### 4.4 Conclusions

In stable CAD patients, combined extreme levels of thromboinflammatory biomarkers, i.e., low ADAMTS13 Ag, high VWF and high CitH_3_ or dsDNA, respectively, predicted MI, non-hemorrhagic stroke, and all-cause mortality despite medication and treatment. The investigated combined biomarker profile may aid in identifying high-risk individuals, and targeting these pathways may become relevant to new treatment strategies. Our study refines risk stratification in stable CAD and creates potential for personalized antithrombotic therapy targeting thromboinflammation.

## Data Availability

The raw data supporting the conclusions of this article will be made available by the authors, without undue reservation.
