# Supplementary material for: Association between neutrophil extracellular traps, the Von Willebrand factor axis, and clinical outcome in stable coronary artery disease
Source: Front Cardiovasc Med. 2026 Feb 26;13:1759117. doi: 10.3389/fcvm.2026.1759117 (PMC12979422; doi:10.3389/fcvm.2026.1759117)
Supplement: Supplementary file 1 [file Datasheet1.docx]

Supplementary Material

**Supplementary Table S1** Levels of CitH_3_ according to clinical characteristics and cardiovascular risk factors at baseline.

|  | CitH_3_ (ng/mL) |
| --- | --- |
| Male (n= 782) | 2.44 (1.57, 4.64) |
| Female (n= 218) | 2.51 (1.49, 4.54) |
| *p-value* | *0.981* |
| Age> mean (n= 500) | 2.35 (1.50, 4.39) |
| Age< mean (n= 500) | 2.57 (1.62, 4.82) |
| *p-value* | ***0.046*** |
| Previous MI (n= 36) | 2.03 (1.47, 3.91) |
| No MI (n= 964) | 2.47 (1.56, 4.68) |
| *p-value* | *0.111* |
| Hypertension (n= 556) | 2.48 (1.63, 4.71) |
| No hypertension (n= 443) | 2.40 (1.46, 4.51) |
| *p-value* | *0.066* |
| Smoke yes (n= 203) | 2.45 (1.52, 4.96) |
| Smoke no (n= 796) | 2.46 (1.57, 4.56) |
| *p-value* | *0.666* |
| Diabetes yes (n= 200) | 2.57 (1.51, 4.58) |
| Diabetes No (n= 800) | 2.44 (1.57, 4.64) |
| *p-value* | *0.643* |
| BMI > mean (n= 469) | 4.03 ±4.29 |
| BMI < mean (n= 522) | 3.78 ±4.45 |
| *p-value* | ***0.022*** |

Statistically significant *p* –values are bolded. Mean age was 62 ± 9 years and mean BMI was 27.4 ±3.7 kg/m^2^.

Abbreviations: CitH_3_ citrullinated histone H3, MI myocardial infarction, BMI body mass index


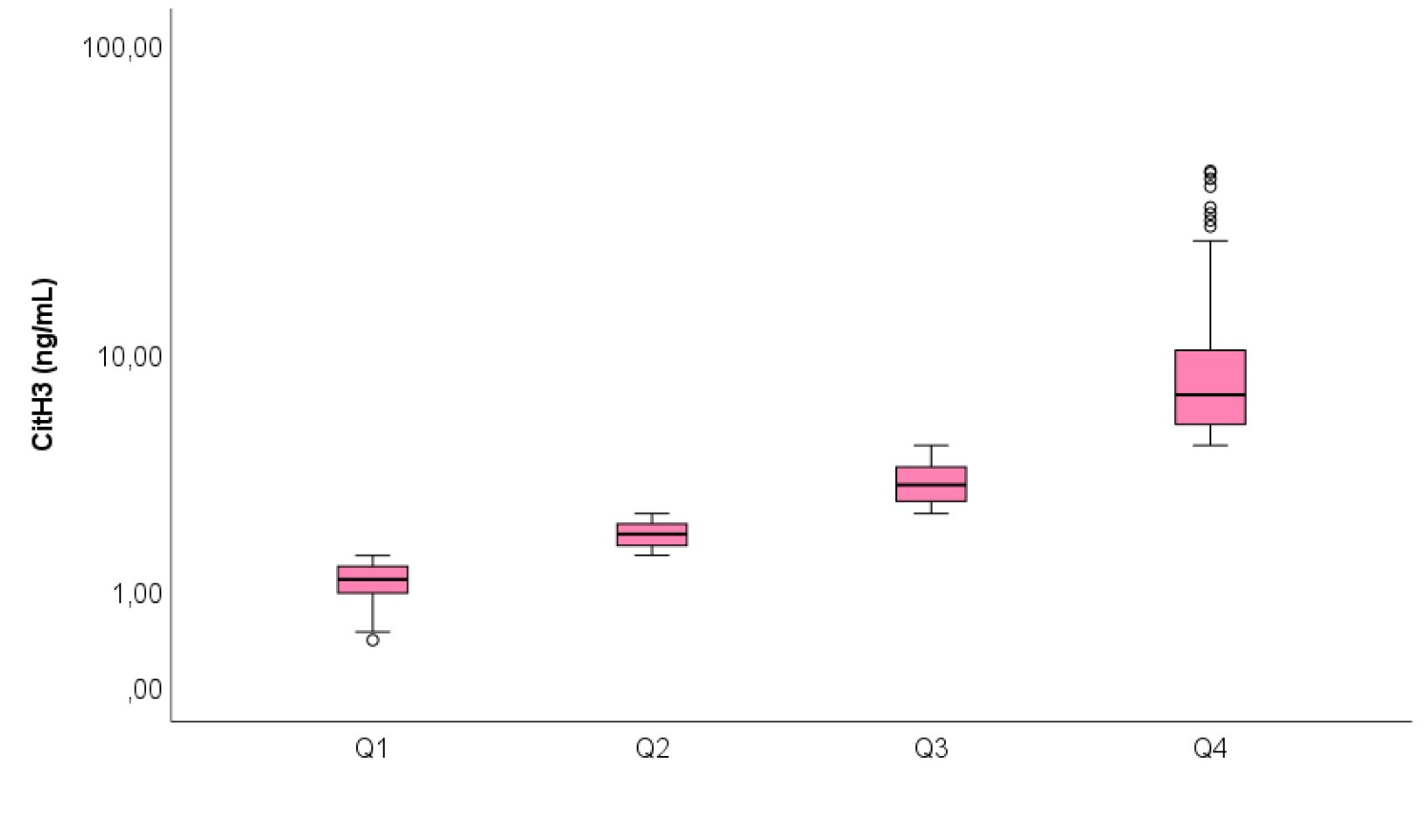


**Supplementary Figure S1** Levels of CitH_3_ in quartile 1 to 4.

Abbreviations: CitH_3_ citrullinated histone H_3,_ Q quartile

**Supplementary Table S2** Univariate logistic regression analyses of the VWF-ADAMTS13 axis alone and combined with NETs biomarkers for the risk of clinical endpoint during 2-year follow-up.

| Biomarker combinations | OR | CI | *p* value |
| --- | --- | --- | --- |
| VWFQ4/ADAMTS13 AgQ1 (sensitivity analysis) | 2.34 | 1.20, 4.54 | 0.012 |
| VWFQ4/ADAMTS13AgQ1/CitH_3_Q4 (combination I) | 3.30 | 1.07, 10.14 | **0.037** |
| VWFQ4/ADAMTS13AgQ1/dsDNAQ4 (combination II) | 4.52 | 1.73, 11.77 | **0.002** |
| VWFQ4/ADAMTS13AgQ1/MPO-DNAQ4 (combination III) | 1.98 | 0.44, 8.95 | 0.375 |

Statistically significant *p* –values are bolded. The sensitivity analysis included the highest quartile of VWF (Q4) and the lowest quartile of ADAMTS13 Ag (Q1). In combination I, II and III the highest quartile of CitH_3_ (Q4), dsDNA (Q4) and MPO-DNA (Q4), respectively, were added.

Abbreviations: NETs neutrophil extracellular traps, VWF Von Willebrand Factor, ADAMTS13 a disintegrin and metalloprotease with thrombospondin type 1 motif member 13, Ag antigen, Q quartile, CitH_3_ citrullinated histone H_3_, dsDNA double stranded DNA, OR odds ratio, CI confidence interval
